# Efficacy and Safety of Single Low Dose Intravenous Fentanyl in Pain Reduction of Lumbar Puncture in Near Term Neonates by A Randomized Clinical Trial

**Published:** 2016

**Authors:** Razieh FALLAH, Samaneh HABIBIAN, Mahmood NOORI-SHADKAM

**Affiliations:** 1 Pediatric Neurologist, Department of Pediatrics, Children Growth Disorders Research Center, Shahid Sadoughi University of Medical Sciences, Yazd, Iran; 2 Pediatrician, Bafgh, Yazd, Iran; 3 Neonatologist, Department of Pediatrics, Shahid Sadoughi University of Medical Sciences, Yazd, Iran

**Keywords:** Intravenous Fentanyl, Lumbar Puncture, Neonate, Pain, Analgesic

## Abstract

**Objective:**

Reduction of pain of invasive procedures in neonates can prevent pain side effects. The purpose of present study was to evaluate the efficacy and safety of a single low dose of intravenous fentanyl in reducing of lumbar puncture (LP)

pain in neonates.

**Materials & Methods:**

In this randomized clinical trial, registered with code number of 2014022616761N150, admitted neonates to Shahid Sadoughi Hospital, Yazd, Iran from August-April 2012 (45 cases) were randomly assigned into two groups to receive 2 μg/kg of intravenous fentanyl or 0.2 milliliter of normal saline, two min before LP. Primary outcome was success rate in reducing of pain during needle insertion to skin (pain score of less than three). Secondary outcomes were clinical side effects and serious adverse events.

**Results:**

Forty-five neonates including 23 girls and 22 boys were evaluated. Pain reduction was obtained in 39.1% (9 of 23 neonates) of fentanyl group and in 4.5% (one of 22 neonates) of control group. Means of pulse rate (136.41± 9.16 vs. 148.9± 8.99) and pain score during needle insertion (3.41±1.31 vs. 5.8±1.12) were lower in fentanyl group. No severe adverse effects were seen in both groups. Side effects such as vomiting [9% (N=2) in control and 4.3% (N=1) in fentanyle group] and mild transient decrease in oxygen saturation in 8.7% (N=2) of fentanyle group were seen. Safety in two groups was not statistically different.

**Conclusion:**

Intravenous fentanyl might be considered as a safe and effective analgesic drug in LP in neonates.

## Introduction

Lumbar puncture (LP) and cerebrospinal fluid (CSF) analysis is essential in confirming the diagnosis of central nervous system infections and is often helpful in assessing of subarachnoid hemorrhage, demyelinating, degenerative diseases and intracranial neoplasms (1).

Comfort and immobility of patients are critical to the success of the procedure. If the child is not cooperative, procedural sedation should be used to reduce child pain and anxiety and to improve kids and their parents’ experience (2, 3).

Different analgesics and sedative drugs such as topical anesthetic agents (1% lidocaine, EMLA cream) (4), propofol and ketamine (5), nitrous oxide (6), oral midazolam and oral promethazine (7), intravenous fentanyl (8) have been used by physicians to reduce pain and stress of LP. However, in spite of progress in the efforts done for management and reduction of pain in children, they are still not receiving satisfactory LP pain control and more improvement can still be gained (9).

Newborns experience pain and stress (10) and pain in premature neonates can alter hypothalamic–pituitary–adrenal axis and cortisol secretion regulation. Higher neonatal procedural pain exposure in less than 28 wk gestational age newborn was related to lower cortisol response to stress, besides, high cortisol levels in infancy may help to cause greater risks of impaired neurodevelopment and less attention (11).

Pain is the most common and severe signs in admitted newborns to neonatal intensive care unit (NICU). 

Measurement for reduction, prevention and management of pain in neonates during routine care or invasive procedures by combination of non-pharmacologic and pharmacologic therapies should be provided to reduce short-term and long-term side effects of pain (12). 

Opioids such as morphine and fentanyl, which have analgesic and sedative effects, a wide therapeutic window, and attenuate responses of physiologic stress, are the most commonly used drugs for moderate to severe pain in neonates (13).

Fentanyl is a rapid-acting opioid analgesic that in comparison to morphine is accompanied by less sedative or hypotensive effects, reduced effects on gastrointestinal motility or urinary retention (14).

There are a few randomized clinical trials compared the efficacy and safety of fentanyl in placebo in control of pain of invasive procedures in neonates. The purpose of present research was to evaluate the efficacy and safety of bolus dose of intravenous fentanyl in reducing of pain of LP in neonates.

## Materials & Methods

A parallel single-blinded randomized clinical trial was conducted on 45 admitted neonates to NICU of Shahid Sadoughi Hospital, Yazd, Iran children who received LP to roll up meningitis from August to April 2012.

The registration number of the research in clinical trial website of Iran is 2014022616761N1.

Informed consent was taken from neonates parents before administration of the drugs and the Ethics Committee of Shahid Sadoughi University of Medical Sciences, Yazd, Iran, approved this clinical trial. 

To determine a 20% difference in efficacy between the two groups, with type one error (alpha) of 0.05 and 80% power, sample size was estimated as 24 neonates in each group.

Eligible participants included NICU admitted neonates with gestational age of more than 34 wk, birth weight of more than 1800 gr and those who underwent lumbar puncture based on clinical judgment of the neonatologist of research.

Those with major congenital anomalies, severe hypoxic ischemic encephalopathy, neuromuscular disorders, hemodynamic or respiratory instability, aged more than two months, decrease in the level of consciousness (Glasgow Coma Scale less than 10), used sedative or analgesic drugs 12 h before LP or whose parents refused to consent, and were excluded. Simple randomization of the study was computer generated by random numbers and allocation ratio was 1:1 for the three groups.

The nurse of research, not involved in the trial, did the randomisation and blinding. Data gatherers, outcome assessors and data analysts were all allocation blinded.

A trained NICU nurse was in charge of allocating each neonate in the randomized treatment group, and she guaranteed that the two preparations would not differentiate. The drugs were delivered by a nurse and primary and secondary outcomes were assessed by the pediatric resident of research who had no information of the sedation regimens group assignment. 

Fifty consecutive neonates who had inclusion criteria of research were randomly assigned to two groups to receive 2 μg/kg of intravenous (IV) fentanyl (ampoule of 0.5 mg in 10 milliliter of Caspian Tamin pharmaceutical Co, Iran) diluted to 0.2 ml of normal saline (Group I) or 0.2 ml of normal saline (Samen pharmaceutical Co, Iran) as placebo (Group II).

In both groups, the drugs were given intravenously two min before undergoing of LP and LP was performed with a trained and experienced pediatric resident, a similar needle size and in seated position. 

Pulse rate, respiratory rate, blood pressure, and saturation of oxygen (SaO2) were measured two min before drugs administration and when the needle was inserted and then pulse oximetry continued and oxygen saturation continuation were measured every two min during the first 10 min after LP by the pediatric resident of research. 

Obtaining pain score of three based on Neonatal Infant Pain Scale or NIPS (15) during needle insertion to skin was considered as sufficient analgesia. 

Primary outcome was success rate in reducing of pain when the needle was inserted to skin for LP (pain score of less than three). Secondary outcomes were clinical adverse events and severe side effects. 

Apnea and assisted ventilation respiratory depression, cyanosis and SaO2 of less than 70%, significant and persistent hypotension (systolic arterial pressure of less than 70 mmHg or 30% or greater decrease in before sedative drug taking mean arterial blood pressure for more than 60 sec), bradycardia (pulse rate less than 60 in min), arrhythmia, seizure and chest wall rigidity, were considered as severe adverse events. 

The data were analyzed using SPSS 17 statistical software (Chicago, IL, USA). Recorded data were assessed for normal distribution using the Kolmogorov-Smirnov test and Chi-square test was used for data analysis of qualitative variables and mean values were compared using independent t-test. P values of less than 0.05 were taken as significant different. 

## Results

**Fig1:**
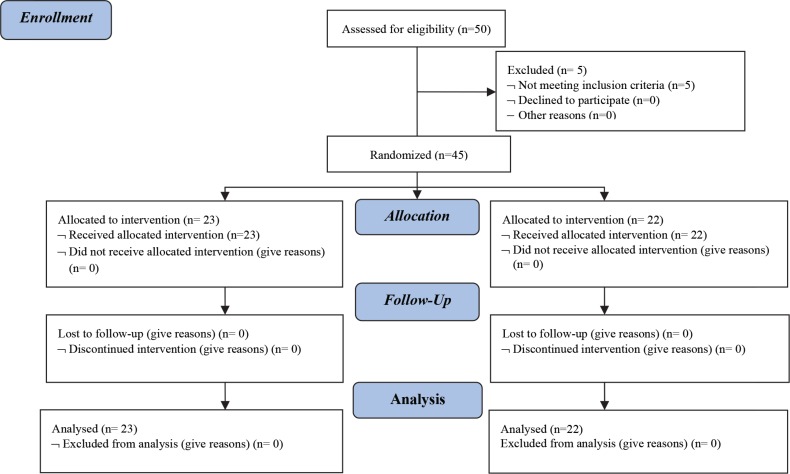
CONSORT 2010 Flow Diagram

In group 1, two neonates with severe hypoxic ischemic encephalopathy and in group 2, one neonate with neuromuscular disorders and two neonates whom received phenobarbital, were excluded and finally 45 neonates including 23 girls (51%) and 22 boys (49%) were evaluated (Figure 1).

By Kolmogorov-Smirnov test, the data had normal distribution.

Table1 shows comparison of demographic characteristics, rout of delivery and pulse rate and oxygen saturation before drug administration in neonates of the two groups which indicated that sex distribution, rout of delivery, mean of age, mean of weight, mean of gestational age, mean of pulse rate and oxygen saturation before drug administration were not statistically significant different in two groups.

Table 2 compares mean of pulse rate during needle insertion and mean of oxygen saturation during, two, four, six, and eight min after drug administration in the neonates of two groups, which shows that neonates in IV fentanyl group had lower pulse rate and pain score during needle insertion. Saturation of oxygen drug administration of two, four, six, eight and ten min after drug administration were not statistically significant different in both groups. 

Pain reduction or achieving the pain score of less than three was obtained in nine neonates (39.1%) in intravenous fentanyl (95% confidence interval: 19.16% to 59.04%) and in one neonate (4.5%) in control (95% confidence interval: 4.16% to 13.16%) groups, respectively. 

Statistical analysis showed that intravenous fentanyl was an effective drug in reduction of lumbar puncture pain in neonates (P= 0.001). No life-threatening or serious side effect was seen in both groups.

Clinical adverse effects such as vomiting [9% (N=2) in control group and 4.3% (N=1) in intravenous fentanyle group] and mild transient decrease in oxygen saturation (SaO2 =85%) immediately corrected by oxygen therapy in 8.7% (N=2) of fentanyle group were seen. 

No statistically significant differences were seen from viewpoint of safety between the two groups.

## Discussion

Prevention and control of pain in neonates that includes routine assessments of pain, decreasing the number of painful procedures, use of pharmacologic and nonpharmacologic techniques in routine minor procedures, treatment of surgery, invasive procedures, pain, avoidance of chronic pain and stress during neonatal intensive care, has short-term and long-term benefits (12).

Insufficient relief of children’s procedural pain and distress affects the experience of them as well as their parents and has adverse impacts on procedural success as well (16).

LP is one of the most frequent painful diagnostic procedures historically been done without local anesthesia, specifically in neonates (9). In Italy, only in 58% of NICU admitted neonates analgesia was used before LP (17). However, use of sedation drugs before LP can reduce incidence of traumatic LP (18). 

Intravenous fentanyl can be used in neonates that provides rapid analgesic effect with the least hemodynamic effects. In some studies, fentanyl has been used for achieving analgesia prior to tracheal intubation in preterm and term newborns and in ventilated preterm neonates. (13, 19, 20) Today, nitrous oxide is used for induction of sedation and analgesia in minor pediatric procedures such as LP and laceration repair. However, the drug might be associated with clinical seizure activity (21).

Our results showed that single dose of 2 μg/kg of intravenous fentanyl can be effective in reducing of pain of LP in term NICU admitted neonates.

Combination of fentanyl and propofol (22) in addition to fentanyl and midazolam (23) for invasive procedures in children with acute leukemia (22, 23) or lymphoma (22) reduced child movement.

In Italy, administration of 1-2 μg/kg of fentanyl in remature neonates before heel lancing provided a more analgesic effects and reduction in the pain score than facilitated tucking (24).

In the present study, administration of intravenous in fentanyl attenuated more heart rate increased than in control group. In another Iranian study, IV fentanyl attenuated more heart rate increased than sufentanil, alfentanil or remifentanil and hemodynamic stability was better kept with fentanyl (25).

In the present study, a single low dose of IV fentanyl in ≥ 34 wk gestational age neonates was safe and life threatening and severe adverse events were not seen in any neonates. Combination of low dose of IV fentanyl and midazolam was effective and safe in reduction of LP pain in 2-7 yr old children (26). However, chest wall rigidity is one of serious side effects of fentanyl and other synthetic opioids, especially, if the drug is administered with rapid and high doses and in neonates and infants (27).

In conclusion, a single low dose of 2 μg/kg of intravenous fentanyl two min before Lp of near term neonates is safe and effective in reduction of this procedural pain. Results of this research can be viewed as being promising and future clinical trials with larger sample size study should be performed for definite proof of fentanyl. 

**Table 1:** Comparison of Demographic Characteristics, Rout of Delivery and Pulse Rate and Oxygen Saturation before Drug Administration in Neonates of the Two Groups

**Data**		**Normal saline**	**Intravenous fentanyl**	***P*** **. value**
**Sex**	**Female (%)**	11(50)	12 (47)	0.6
	**Male (%)**	11 (50)	11 (53)	
**Rout of delivery**	**Vaginal (%)**	10 (45.5)	11 (47)	0.9
	**Cesarean section (%)**	12 (54.5)	12 (53)	
**Age in days (yr) (mean ±SD)**		28.61 ± 11.32	29.14± 10.64	0.8
**Weight in grams (mean ±SD)**		3312.7 ± 892.1	3360.8 ± 934.3	0.8
**Gestational age in weeks (mean ±SD)**		37.6 ± 0.6	37. 4± 0.8	0.3
**Pulse rate before drug administration (mean ±SD)**		137.1 ± 9.83	134.81 ± 9.31	0.4
**Oxygen saturation before drug administration (mean ±SD)**		96.31 ± 2.11	95.87 ± 2.65	0.7

**Table 2:** Comparison of Mean of Pulse Rate During Needle Insertion and Mean of Oxygen Saturation During, 2, 4, 6 and 8 Minutes after Drug Administration in The Neonates of Two Groups

**Groups**	**Normal saline** **(mean ±SD)**	**IV fentanyl** **(mean ±SD)**	***P*** **. value**
**Data**			
**Pulse rate during needle insertion**	148.9 ± 8.99	136.41 ± 9.16	0.001
**Oxygen saturation during drug administration**	96.11 ± 2.23	96.21 ±1.81	0.8
**Oxygen saturation two minutes after drug administration**	95.82 ± 2.25	94.51± 2.87	0.9
**Oxygen saturation four minutes after drug administration**	94.86 ± 2.51	94.52 ± 3.34	0.7
**Oxygen saturation six minutes after drug administration**	96.11 ± 2.31	95.18 ± 2.84	0.9
**Oxygen saturation eight minutes after drug administration**	95.76 ± 2.36	96.31 ± 2.55	0.7
**Oxygen saturation ten minutes after drug administration**	96.31 ± 2.46	96.25 ± 2.78	0.9
**Pain score during needle insertion**	5.8 ± 1.12	3.41± 1.31	0.001
